# Assessing the impact of a mushroom-derived food ingredient on vitamin D levels in healthy volunteers

**DOI:** 10.1186/s12970-020-00387-0

**Published:** 2020-11-11

**Authors:** Jorge Marques Pinto, Viviane Merzbach, Ashley G. B. Willmott, Jose Antonio, Justin Roberts

**Affiliations:** 1 Cambridge Centre for Sport and Exercise Sciences, School of Psychology and Sport Science, Anglia Ruskin University, Compass House, East Road, Cambridge, CB1 1PT UK; 2 Exercise and Sport Science, Nova Southeastern-Florida University, Davie, FL USA

**Keywords:** Vitamin D status, Vitamin D_2_, Recreationally active, UV radiated mushrooms

## Abstract

**Background:**

Prevalence of vitamin D insufficiency/deficiency has been noted in athletic populations, although less is known about recreationally active individuals. Biofortification of natural food sources (e.g. UV radiated mushrooms) may support vitamin D status and is therefore of current scientific and commercial interest. The aim of this study was to assess the impact of a mushroom-derived food ingredient on vitamin D status in recreationally active, healthy volunteers.

**Methods:**

Twenty-eight participants were randomly assigned to either: 25 μg (1000 IU) encapsulated natural mushroom-derived vitamin D_2_; matched-dose encapsulated vitamin D_3_ or placebo (PL) for 12 weeks. Venous blood samples were collected at baseline, week 6 and 12 for analysis of serum 25(OH)D_2_ and 25(OH)D_3_ using liquid chromatography mass spectrometry. Habitual dietary intake and activity were monitored across the intervention.

**Results:**

Vitamin D status (25(OH)D_TOTAL_) was significantly increased with vitamin D_3_ supplementation from 46.1 ± 5.3 nmol·L^− 1^ to 88.0 ± 8.6 nmol·L^− 1^ (*p* < 0.0001) across the intervention, coupled with an expected rise in 25(OH)D_3_ concentrations from 38.8 ± 5.2 nmol·L^− 1^ to 82.0 ± 7.9 nmol·L^− 1^ (*p* < 0.0001). In contrast, D_2_ supplementation increased 25(OH)D_2_ by + 347% (7.0 ± 1.1 nmol·L^− 1^ to 31.4 ± 2.1 nmol·L^− 1^, *p* < 0.0001), but resulted in a − 42% reduction in 25(OH)D_3_ by week 6 *(p* = 0.001). A net + 14% increase in 25(OH)D_TOTAL_ was established with D_2_ supplementation by week 12 (*p* > 0.05), which was not statistically different to D_3_. Vitamin D status was maintained with PL, following an initial − 15% reduction by week 6 (*p* ≤ 0.046 compared to both supplement groups).

**Conclusions:**

The use of a UV radiated mushroom food ingredient was effective in maintaining 25(OH)D_TOTAL_ in healthy, recreationally active volunteers. This may offer an adjunct strategy in supporting vitamin D intake. However, consistent with the literature, the use of vitamin D_3_ supplementation likely offers benefits when acute elevation in vitamin D status is warranted.

## Introduction

Vitamin D in its two most common forms, ergocalciferol (vitamin D_2_) and cholecalciferol (vitamin D_3_), is a pro-hormone [1] involved in numerous physiological processes including: bone mineralisation, calcium and phosphorus homeostasis, neuromuscular function, cell growth regulation and immune modulation [2–5]. Both forms of vitamin D undertake the same enzymatic hydroxylation reactions to become biologically active. The first reaction takes place in the liver catalysed by the action of 25-hydroxylase, which converts vitamin D_2_ or D_3_ to 25-hydroxyvitamin D_2_ (25(OH)D_2_) and 25-hydroxyvitamin D_3_ (25(OH)D_3_), respectively. Following transport to the kidneys by vitamin D-binding proteins (DBP) and further catalysation by 1-α-hydroxylase, both forms are converted into active 1,25-dihydroxyvitamin D (1,25(OH)_2_D) [4]. It has been shown that both 1,25(OH)_2_D_2_ and 1,25(OH)_2_D_3_ have similar affinities for the vitamin D receptor (VDR) [6, 7], and comparably influence biological activity in vivo [8].

Modulation of vitamin D concentrations occurs through endogenous synthesis following ultra-violet (UV) sunlight radiation exposure (wavelengths 290–315 nm) and resulting conversion of 7-dehydrocholesterol to vitamin D_3_ [4]. In the Northern hemisphere (latitudes of > 30° north), or where exposure to such UV radiation is limited (particularly across autumn/winter periods), vitamin D insufficiency (25(OH)D_TOTAL_ level < 50 nmol·L^− 1^) [9] can have health implications which may go unrecognised [10]. Indeed, a recent UK nutrition survey reported vitamin D deficiency (< 25 nmol·L^− 1^) in 15% of women and 19% of men aged 19–64 years [11]; with other authors highlighting that only 18 and 24.1% of women and men in the UK, respectively, were classed as having ‘adequate’ vitamin D status [12, 13]. Worldwide it is estimated that approximately 1 billion people are considered to have vitamin D insufficiency or deficiency (25(OH)D_TOTAL_ < 50 nmol·L^− 1^) [2]. Previous research has also demonstrated that trained athletes may be at risk of vitamin D insufficiency or deficiency [14, 15], which can impact on training adaptations, exercise recovery and injury prevalence [16, 17], and should be regularly monitored. However, less is known about recreationally active individuals who may also be at a similar risk of lowered vitamin D status.

Vitamin D status can also be influenced by dietary intake, with animal sources (e.g. cod liver oil, salmon, cheese, red meats, milk, eggs) [18] and fortified foods providing exogenous vitamin D_3_; and plant- or fungi-based foods (e.g. phytoplankton, mushrooms, yeast) providing small quantities of vitamin D_2_. According to the Scientific Advisory Committee on Nutrition (UK) [19], the average daily intake necessary to sustain 25(OH)D_TOTAL_ levels above 25 nmol·L^− 1^ during the winter season in the UK is ~ 10 μg·d^− 1^ (400 IU·d^− 1^), with average dietary intakes reportedly lower than this [11]. As such, food-based solutions and supplementation to increase vitamin D intake in the population have been strongly emphasised [20].

Whilst fortification offers one potential solution, the lack of diversity of food items has been suggested as a reason for relatively low overall contribution to vitamin D intake [11]. Supplementation with vitamin D_3_ offers another effective strategy to increase dietary intake and raise physiological concentrations of vitamin D [21]. However, costly synthetic production and the sources used (e.g. lanolin and fish oil) [22, 23] potentially make these strategies impractical or unsuitable for specific dietary regimes (e.g. vegan/vegetarian) [12]. Biofortification offers a new approach to increasing the nutritional content of a wide range of foods, supporting dietary requirement inclusivity [4, 24]. As example, a new method of UV radiation of edible mushrooms [24] has the potential to produce more bioavailable vitamin D_2_ at relatively low cost [20, 23, 25–27], with specific species (i.e. *Agaricus bisporus*, *Lentinula edodes* and *Pleurotus ostreatus*) achieving up to 40 μg of ergocalciferol per 100 g of dried mushroom mass [10, 23].

It has been suggested that the bioefficacy between vitamin D_2_ and D_3_ differs [28], with intervention studies highlighting a superior effect of vitamin D_3_ in raising 25(OH)D_TOTAL_ levels [29–42]. However, other studies contest there is less difference in the bioefficacy of vitamin D_2_ compared to D_3,_ especially when supplementation administered as daily dosages is considered [21, 29, 32, 35, 38, 40, 43–45]. Few studies have investigated the impact of vitamin D_2_ supplementation from natural sources (e.g. UV radiated mushrooms) on vitamin D status [24], and the heterogeneity of those studies (e.g. non-placebo control, variable dosages, population type) makes it difficult to draw meaningful conclusions on whether vitamin D_2_ derived from mushrooms was effective. As such, the aim of this study was to conduct an independent assessment into the impact of a commercially utilised mushroom-derived food ingredient on vitamin D status in recreationally active healthy volunteers, compared with both vitamin D_3_ and placebo-control supplementation. It was hypothesised that natural vitamin D_2_ would provide an adjunct strategy to support vitamin D status compared with vitamin D_3_.

## Methods

### Study design

This study employed a randomised, double-blinded, placebo-controlled design over a 12-week period. The study was conducted in accordance with the Declaration of Helsinki (2013), with ethical approval from the Faculty of Science and Technology Ethics Committee, Anglia Ruskin University (Project Number: FST/FREP/18773).

### Participants

An a priori power calculation based on previous data [36] utilising α = 0.05 and 1-β = 0.8, estimated a sample size of 27 participants. Following a study briefing, all participants provided written informed consent prior to study inclusion. Participants were required to be healthy volunteers, satisfactorily complete a health screen questionnaire, and be prepared to comply with study requirements. Participants with a known history of cardio-metabolic disorders, blood related disorders, and recent viral infections were not eligible for study inclusion. Likewise, anyone reporting use of prescribed medication or supplementation (e.g. current vitamin D use) which could conflict with the study parameters, as well as those with known adverse or allergic reactions to dietary intake of mushrooms were not included in the study. Based on the nature of the supplementation, vegans were also not eligible for study inclusion. Any participants with high starting vitamin D levels (> 150 nmol·L^− 1^) were not included in the study.

Thirty-three participants (20 males, 13 females) were initially recruited. One participant was subsequently withdrawn due to medication use conflicting with study parameters; three participants were withdrawn due to non-compliance with food/activity diaries, and data for one participant was excluded due to high initial starting vitamin D concentration based on recent use of vitamin D_3_ supplementation. Twenty-eight participants (16 males, 12 females) were therefore included in the final analysis having completed all aspects of the study. Participant characteristics are shown in Table 1.

**Table 1:** Characteristics of participants at baseline by intervention group

	Vitamin D_**2**_ (*n* = 10, 7 M, 3 F)	Vitamin D_**3**_ (*n* = 10, 5 M, 5 F)	PL(*n* = 8, 4 M, 4 F)
Age (yrs)	36 ± 3	38 ± 4	30 ± 3
Height (cm)	174.4 ± 3.1	171.8 ± 2.1	173.2 ± 4.3
Body mass (kg)	74.0 ± 3.7	78.2 ± 5.0	77.5 ± 6.7
Body fat (%)	22.2 ± 2.4	27.9 ± 3.6	25.2 ± 4.2
Body mass index (kg·m^2^)	24.4 ± 1.3	26.6 ± 1.8	25.4 ± 1.2

*M* male, *F* female, *PL* placebo. No differences reported between groups for any variable. Data are presented as mean ± standard error (M ± SE)

### Procedures

All testing procedures took place in the Cambridge Centre for Sport and Exercise Sciences laboratories at Anglia Ruskin University, under controlled conditions between January–April 2019. Participants were required to attend the laboratory, having rested in the 24-h prior and having had their last meal ~ 12-h before the appointment, at baseline, week 6 and week 12. Upon arrival, each participant’s height was measured using a stadiometer (Seca CE123, Hamburg, Germany), and body mass and body fat percentage were assessed through the use of bioelectrical impedance analysis scales (Tanita BC420SMA, Amsterdam, The Netherlands).

#### Blood sampling and analysis

Once anthropometric measurements were recorded, participants rested in a semi-prone position for 5-min prior to a venous whole blood sample collection by a qualified phlebotomist into duplicate 4 mL K3EDTA vacutainers (Greiner Bio-One GmbH, Kremsmunster, Austria). Samples were centrifuged for 10-min at 2000 rpm, with aliquoted serum pipetted into sterile, non-pyrogenic, polypropylene cryovials (Fisherbrand, Fisher Scientific, Loughborough, UK) and frozen at − 20 °C for later assessment of serum 25(OH)D_2_ and 25(OH)D_3_. All samples were analysed in conjunction with the Core Biochemical Analysis Laboratory (CBAL), Addenbrookes Hospital, Cambridge. Liquid chromatography-mass spectrometry (AB Sciex Mass spectrometer [API5500]) was utilised for the quantitative analysis of 25(OH)D_2_ and 25(OH)D_3_. The lower quantitation limit for the assay was 5 nmol·L^− 1^ for both 25(OH)D_2_ and 25(OH)D_3_, and the upper limit was 130 nmol·L^− 1^ and 170 nmol·L^− 1^ for 25(OH)D_2_ and 25(OH)D_3_, respectively [46].

#### Supplement interventions

Following baseline assessment, participants were category-coded according to initial vitamin D levels (e.g. deficient < 25 nmol·L^− 1^, insufficient 25–49 nmol·L^− 1^, inadequate 50–74 nmol·L^− 1^, adequate > 75 nmol·L^− 1^) [47], and then within category randomly assigned to intervention condition to minimise testing bias. As such, participants were allocated in a double-blinded manner to one of the three intervention groups. At baseline and week 6 visits, participants were provided (according to their initial intervention group allocation) with a 6-week supply of either: encapsulated vitamin D_2_ (VitaShroomD, Cambridge Commodities Ltd. [CCL]), containing 25 μg (1000 IU) of natural mushroom-derived vitamin D_2_ powder; encapsulated vitamin D_3_ (Cholecalciferol, CCL), containing 25 μg (1000 IU) of vitamin D_3_, or placebo (PL, ProEarth Organic Sunflower Protein 45%, CCL). All products were manufactured and pre-capsulated (hypromellose vegetable capsules) to clinical standards via CCL and evaluated by the European Food Safety Authority. All supplements were provided in standardised opaque sealed pots for hygiene and double-blinding purposes and administered independently of the manufacturing company. As a means to monitor supplement adherence, participants were required to complete a daily compliance record throughout the intervention. As a cross-check measure, participants returned pots at follow-up visits, and excess capsules were counted.

#### Dietary intake and activity monitoring

All participants were required to complete food and activity diaries to assess individual consistency across the intervention period. At the baseline visit, participants were provided with an individual MyFitnessPal account to record their dietary intake and were instructed to maintain their physical activity levels and dietary habits throughout the intervention period. For exercise activity across the intervention, participants recorded exercise type, duration, and overall session rating of perceived exertion (sRPE), with estimated training load, monotony, and strain determined as previously described [48, 49]. Food diaries were collated by participants in the first 7-days of supplementation and the 7-days leading into the week-6 and week-12 laboratory visits, respectively. Participants were provided with example diaries and individually instructed in diary completion, with emphasis on meal breakdown, portion size/weight and weighing procedure. Dietary analyses were undertaken by the same researcher for standardisation by transferring data for three weekdays and one weekend day from the individual MyFitnessPal accounts to the Nutritics Professional Dietary Analysis software (Nutritics Limited, Dublin), utilising the Composition of Foods Integrated Dataset (COFIDS) incorporating McCance and Widdowson (7th Edition) database.

### Statistical analysis

Statistical analyses were performed using SPSS (IBM, Version 24.0). Normality of data was verified by the Shapiro-Wilk test. Outliers were identified by inspection of box plots > 1.5 IQR in SPSS. Baseline measures were assessed using between groups ANOVAs. Repeated measures ANOVAs were used to compare group x time effects with Bonferroni post-hoc assessment where applicable. Where sphericity was violated a Greenhouse-Geisser correction was applied. An alpha level of *p* ≤ 0.05 was considered statistically significant for all tests. Data are presented as M ± SE.

## Results

### Dietary intake, supplement compliance and activity monitoring

Mean dietary intakes at baseline and across the intervention are shown in Table 2 (absolute) and Table 3 (relative). Energy intake was initially 30% higher (+ 534 kcal·d^− 1^) at baseline for D_2_ compared to D_3_ only (*p* = 0.03). This corresponded with a 56% higher (+ 96 g·d^− 1^) carbohydrate intake for D_2_ compared with D_3_ only (*p* = 0.006), and similarly, was reflected in relative intakes at baseline. For main macronutrients, no differences were reported between and within groups at either week 6 or 12, highlighting dietary consistency.

**Table 2:** Dietary intake (total) at baseline, week 6 and 12 by intervention group

Variable	Vitamin D_**2**_	Vitamin D_**3**_	PL
*Energy intake (kcal·d*^*− 1*^*)*			
*Baseline*	2397.9 ± 136.0*	1844.3 ± 157.0	1946.4 ± 128.7
*Week 6*	2035.6 ± 132.9	1919.0 ± 181.2	1984.0 ± 226.3
*Week 12*	2123.1 ± 152.6	1724.2 ± 185.3	1824.8 ± 199.5
*Carbohydrate (g·d*^*− 1*^*)*			
*Baseline*	268.3 ± 29.8*	172.1 ± 12.7	202.7 ± 8.6
*Week 6*	226.2 ± 20.9	199.4 ± 26.7	208.0 ± 18.5
*Week 12*	222.0 ± 23.2	180.8 ± 19.9	195.7 ± 21.5
*Fat (g·d*^*− 1*^*)*			
*Baseline*	94.9 ± 4.4	80.2 ± 8.3	80.1 ± 7.3
*Week 6*	80.3 ± 9.6	76.9 ± 6.3	83.6 ± 13.0
*Week 12*	91.2 ± 7.3	75.1 ± 9.5	75.2 ± 9.4
*Protein (g·d*^*− 1*^*)*			
*Baseline*	107.4 ± 11.0	88.3 ± 7.3	102.4 ± 13.4
*Week 6*	95.2 ± 11.9	85.5 ± 9.1	96.0 ± 12.7
*Week 12*	97.9 ± 13.7	69.8 ± 6.6	90.4 ± 14.5
*Calcium (mg·d*^*− 1*^*)*			
*Baseline*	1024.7 ± 124.3*	677.4 ± 50.9	904.4 ± 83.8
*Week 6*	927.1 ± 90.0	693.9 ± 87.8	776.2 ± 87.6
*Week 12*	961.3 ± 110.2	705.3 ± 92.2	608.0 ± 70.8^#^
*Vitamin D (*μg*·d*^*− 1*^*)*			
*Baseline*	4.6 ± 1.0	2.9 ± 0.5	2.9 ± 0.9
*Week 6*	5.3 ± 1.3	3.2 ± 0.9	4.1 ± 0.7
*Week 12*	4.4 ± 1.4	2.6 ± 0.6	3.9 ± 0.9

^*^denominates significant difference to vitamin D_3_ at baseline only (*p* ≤ 0.03). ^#^ denominates significant difference to both baseline and week 6 within group only (*p* ≤ 0.03)

**Table 3:** Dietary intake (relative) at baseline, week 6 and 12 by intervention group

Variable	Vitamin D_**2**_	Vitamin D_**3**_	PL
*Energy intake (kcal·kg*^*− 1*^*·d*^*− 1*^*)*			
*Baseline*	32.6 ± 1.2*	24.3 ± 2.3	26.1 ± 2.2
*Week 6*	28.1 ± 1.9	25.0 ± 1.8	26.0 ± 2.3
*Week 12*	29.3 ± 2.1	22.8 ± 2.3	24.2 ± 1.6
*Carbohydrate (g·kg*^*− 1*^*·d*^*− 1*^*)*			
*Baseline*	3.6 ± 0.3*	2.3 ± 0.2	2.8 ± 0.4
*Week 6*	3.1 ± 0.2	2.6 ± 0.3	2.8 ± 0.3
*Week 12*	3.0 ± 0.3	2.4 ± 0.2	2.7 ± 0.3
*Fat (g·kg*^*− 1*^*·d*^*− 1*^*)*			
*Baseline*	1.3 ± 0.1	1.1 ± 0.1	1.1 ± 0.1
*Week 6*	1.1 ± 0.2	1.0 ± 0.1	1.1 ± 0.1
*Week 12*	1.3 ± 0.1	1.0 ± 0.1	1.0 ± 0.1
*Protein (g·kg*^*− 1*^*·d*^*− 1*^*)*			
*Baseline*	1.5 ± 0.1	1.2 ± 0.1	1.3 ± 0.1
*Week 6*	1.3 ± 0.1	1.1 ± 0.1	1.3 ± 0.1
*Week 12*	1.3 ± 0.1	0.9 ± 0.1	1.2 ± 0.1
*Calcium (mg·kg*^*− 1*^*·d*^*− 1*^*)*			
*Baseline*	14.0 ± 1.6	9.0 ± 1.0	12.6 ± 2.0
*Week 6*	12.7 ± 1.1	9.5 ± 1.5	10.8 ± 1.6
*Week 12*	13.1 ± 1.3	9.5 ± 1.5	8.5 ± 1.1^#^
*Vitamin D (μg·kg*^*− 1*^*·d*^*− 1*^*)*			
*Baseline*	0.06 ± 0.01	0.04 ± 0.01	0.04 ± 0.01
*Week 6*	0.08 ± 0.02	0.04 ± 0.01	0.06 ± 0.01
*Week 12*	0.06 ± 0.02	0.03 ± 0.01	0.05 ± 0.01

^*^denominates significant difference to vitamin D_3_ at baseline only (*p* ≤ 0.01). ^#^ denominates significant difference to both baseline and week 6 within group only (*p* ≤ 0.01)

For selected micronutrients, it was noted that absolute calcium intake was 51% higher (+ 347.3 mg·d^− 1^) for D_2_ compared with D_3_ also at baseline only (*p* = 0.03). A group x time interaction effect was also found for calcium, with absolute intakes (F = 2.99, *p* = 0.028, ηp^2^ = 0.20) being significantly reduced at week 12 for PL only compared with week 6 (*p* = 0.029) and baseline (*p* = 0.001). No other differences were reported between conditions for any of the dietary variables, including vitamin D intake. Average supplement compliance was reported at 93.1 ± 1.5% (with no differences reported between intervention groups: 95.8 ± 1.2% (vitamin D_2_), 91.0 ± 2.2% (vitamin D_3_) and 93.0 ± 4.3% (PL); *p* > 0.05).

Mean weekly activity monitoring is shown in Table 4. For weekly training load, a significant interaction effect was found (F = 5.37, *p* = 0.013, ηp^2^ = 0.34), with post-hoc analysis demonstrating that training load was lower in the second 6 weeks for PL only (*p* = 0.007). However, no differences were reported between groups for training load, monotony and strain, indicating relative consistency in activity patterns across the intervention period.

**Table 4:** Mean physical activity load over weeks 0–6 (T1) and 7–12 (T2) by intervention group

Variable	Vitamin D_**2**_	Vitamin D_**3**_	PL
*Weekly training load (AU)*			
T1	1538 ± 321	1413 ± 627	1954 ± 307
T2	1498 ± 262	1690 ± 617	1313 ± 327*
*Training monotony (AU)*			
T1	1.2 ± 0.2	1.5 ± 0.4	1.0 ± 0.1
T2	1.1 ± 0.1	1.3 ± 0.3	1.1 ± 0.4
*Training strain (AU)*			
T1	2163 ± 621	2900 ± 1541	2037 ± 369
T2	1960 ± 456	2873 ± 1509	1584 ± 694

^*^denominates significant difference within group only (*p* = 0.007). *AU* arbitrary units

### Vitamin D status

**Fig. 1:**
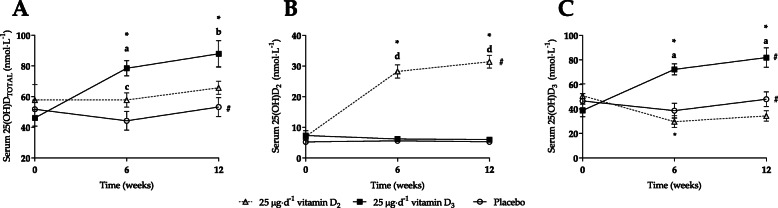
Vitamin D status (absolute) in response to 12-week supplementation intervention. Panels represent: **a**) total serum 25(OH)D, **b**) serum 25(OH)D2 and **c**) serum 25(OH)D3 concentrations taken at baseline, week 6 and week 12, respectively. ^*^ = significant difference within group compared to baseline (*p* ≤ 0.001); ^#^ = significant difference within group compared to week 6 (*p* < 0.05); ^a^ = D_3_ significantly different to D_2_ and PL at timepoint (*p* ≤ 0.01); ^b^ = D_3_ significantly different to PL at timepoint (*p* = 0.006); ^c^ = D_2_ significantly different to PL at timepoint (*p* = 0.046); ^d^ = D_2_ significantly different to D_3_ and PL at timepoint (*p* ≤ 0.001)

At baseline, only 4 participants (14%) were deemed to have ‘desirable’ total vitamin D levels (> 75 nmol·L^− 1^), with 9 (32%) and 14 (50%) participants being categorised as either ‘inadequate’ or ‘insufficient’ levels, respectively [42]. Only 1 individual was recorded as being deficient (4%), with total vitamin D < 25 nmol·L^− 1^. Vitamin D status is shown in Figs. 1 (absolute) and 2 (normalised).

**Fig. 2:**
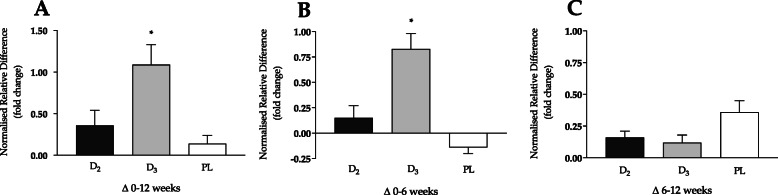
Vitamin D (25(OH)D) status (normalised relative difference) in response to 12-week supplementation intervention. Panels represent: **a**) overall pattern (baseline to week 12), **b**) first 6-week period, and **c**) second 6-week period. ^*^ = significantly different to both D_2_ and PL (*p* ≤ 0.03). Vitamin D measured in nmol·L^− 1^

A significant interaction effect was found for total vitamin D status (25(OH)D_TOTAL_; F = 7.31, *p* = 0.002, ηp^2^ = 0.38), with vitamin D_3_ supplementation resulting in a 70% increase in the first 6 weeks (46.1 ± 5.3 nmol·L^− 1^ to 78.5 ± 5.1 nmol·L^− 1^, *p* < 0.0001), and a further 12% increase to 88.0 ± 8.6 nmol·L^− 1^ (*p* < 0.0001) by week 12. These increases were significantly different to mean values for both vitamin D_2_ and PL at week 6 (*p* ≤ 0.01), but only PL by week 12 (*p* = 0.006). This corresponded with an increase in mean serum 25(OH)D_3_ (interaction effect: F = 16.79, *p* < 0.0001, ηp^2^ = 0.58) for those taking vitamin D_3_, at week 6 (72.3 ± 4.5 nmol·L^− 1^) and week 12 (82.0 ± 7.9 nmol·L^− 1^, *p* = 0.049 compared to week 6, *p* < 0.0001 both compared to baseline). Based on individual adherence rates, it was estimated that vitamin D_3_ supplementation resulted in a + 0.05 ± 0.01 nmol·L^− 1^ mean increase in total vitamin D per 100 IU ingested.

The intake of mushroom-derived vitamin D_2_ resulted in a significant elevation in mean serum 25(OH)D_2_ (interaction effect: F = 71.62, *p* < 0.0001, ηp^2^ = 0.86) from 7.0 ± 1.1 nmol·L^− 1^ to 28.2 ± 2.2 nmol·L^− 1^ by week 6 (*p* < 0.0001), and a further increase to 31.4 ± 2.1 nmol·L^− 1^ by week 12 (*p* = 0.009 compared to week 6), representing an overall change of + 347%. However, this also corresponded with a significant 42% reduction in 25(OH)D_3_ by week 6 (50.8 ± 9.7 nmol·L^− 1^ to 29.6 ± 4.9 nmol·L^− 1^, *p* = 0.001), with only partial recovery (34.4 ± 4.2 nmol·L^− 1^) by week 12 (albeit not significantly different compared to baseline).

As such, vitamin D_2_ supplementation maintained mean vitamin D status (25(OH)D_TOTAL_) across the first 6 weeks, with a 14% increase to 65.8 ± 4.3 nmol·L^− 1^ by week 12 (which was not significantly different to vitamin D_3_). Based on individual adherence rates, it was estimated that vitamin D_2_ supplementation resulted in a + 0.01 ± 0.01 nmol·L^− 1^ mean increase in total vitamin D per 100 IU ingested (*p* = 0.013 compared to vitamin D_3_). Mean vitamin D status was largely maintained with PL over the 12-weeks. However, within condition, an initial, yet non-significant 15% reduction to 44.2 ± 6.2 nmol·L^− 1^ occurred by week 6, which preceded a subsequent increase to baseline values by week 12 (*p* = 0.023). Mean vitamin D status for PL was significantly different to both D_2_ and D_3_ groups at week 6 (*p* ≤ 0.046), but only the D_3_ group by week 12 (*p* = 0.006).

When vitamin D status was expressed as normalised relative difference (Fig. 2), there was an overall main effect reported for mean 25(OH)D_TOTAL_ (F = 6.29, *p* = 0.006). Vitamin D_3_ supplementation resulted in a + 1.09 ± 0.24 normalised increase by week 12, compared with + 0.36 ± 0.18 for vitamin D_2_ (*p* = 0.03) and + 0.14 ± 0.10 nmol·L^− 1^ for PL (*p* = 0.01). This was largely accounted for by a + 1.07 ± 0.23 normalised increase for mean 25(OH)D_3_ (Table 5) in the first 6 weeks with vitamin D_3_ supplementation (F = 29.32, *p* < 0.0001), and a corresponding + 0.83 ± 0.15 normalised increase for mean 25(OH)D_TOTAL_ (F = 16.95, *p* < 0.0001) compared with both vitamin D_2_ and PL (*p* ≤ 0.001). No significant differences were reported between conditions for normalised mean 25(OH)D_3_ (Table 5) or 25(OH)D_TOTAL_ (Fig. 2c) in the final 6 weeks of the intervention (*p* > 0.05).

**Table 5:** Mean normalised relative difference for 25(OH)D_3_ concentrations (fold-change)

	Vitamin D_**2**_	Vitamin D_**3**_	PL
Δ 0–12 weeks	− 0.22 ± 0.09	1.43 ± 0.38 *	0.16 ± 0.11
Δ 0–6 weeks	−0.38 ± 0.04	1.07 ± 0.23*	−0.16 ± 0.06
Δ 6–12 weeks	0.23 ± 0.08	0.13 ± 0.06	0.43 ± 0.11

*denominates significant difference to both vitamin D_2_ and PL groups (*p* ≤ 0.009)

Vitamin D_2_ supplementation resulted in a considerable normalised increase in 25(OH)D_2_ (F = 40.81, *p* < 0.0001, Table 6) in the first 6-weeks (+ 3.55 ± 0.52), but only a small increase of + 0.13 ± 0.05 in the final 6-weeks (F = 5.36, *p* = 0.012), with both responses being significantly different to both vitamin D_3_ and PL (*p* ≤ 0.03). The normalised change in 25(OH)D_TOTAL_ for the vitamin D_2_ group was consistent across both 6-week periods (+ 0.15 ± 0.12 and + 0.16 ± 0.05) (Fig. 2b and c), based on a significant reduction in 25(OH)D_3_ in the first period (− 0.38 ± 0.04, *p* < 0.0001 compared to vitamin D_3_), and a positive (non-significant) gain of + 0.23 ± 0.08 in the second period (*p* > 0.05).

**Table 6:** Mean normalised relative difference for 25(OH)D_2_ concentrations (fold-change)

	Vitamin D_**2**_	Vitamin D_**3**_	PL
Δ 0–12 weeks	4.24 ± 0.69 *	−0.08 ± 0.06	0.00 ± 0.00
Δ 0–6 weeks	3.55 ± 0.52 *	−0.07 ± 0.04	0.06 ± 0.05
Δ 6–12 weeks	0.13 ± 0.05 *	−0.02 ± 0.02	−0.04 ± 0.04

*denominates significant difference to both vitamin D_3_ and PL groups (*p* ≤ 0.03)

## Discussion

The main finding of this study was that 12-weeks supplementation of commercially available vitamin D_3_ significantly increased 25(OH)D_TOTAL_ by 91% in recreationally active participants. This was largely explained by the significant 70% increase, which occurred over the first 6-week period. In contrast, non-significant increases in 25(OH)D_TOTAL_ were reported for the vitamin D_2_ (+ 14%) and PL groups (+ 3%) across the intervention period, largely accounted for in the final 6-weeks of the study. As such, by week 6 vitamin D_3_ supplementation significantly increased 25(OH)D_TOTAL_ compared to both vitamin D_2_ and PL. This was particularly apparent when data was normalised, with vitamin D_3_ demonstrating a + 83% normalised increase, compared to + 15% for vitamin D_2_ and a reduction of − 14% for PL.

By week 12, absolute 25(OH)D_TOTAL_ were only significantly higher in the vitamin D_3_ group compared to PL group, but not to the vitamin D_2_ group. However, when normalised values were considered, the relative increase of + 109% for vitamin D_3_ was significantly greater than both the + 36% and + 14% relative increases observed in the vitamin D_2_ and PL groups, respectively. These findings are in agreement with previous research highlighting the superior effectiveness of daily vitamin D_3_ supplementation compared to commercial vitamin D_2_ in improving vitamin D status [34–36, 40, 42, 43, 50]. Generally, vitamin D_2_ has been shown to be less efficient than vitamin D_3_, however, some research has suggested that vitamin D_2_ supplementation can be effective for maintaining or increasing vitamin D status [21, 34, 35, 42–45]. To our knowledge, there is only one study, which has shown superiority of vitamin D_2_ in comparison to vitamin D_3_ when administered daily [32].

Currently, there are a limited number of studies that have assessed the effects of vitamin D_2_ products derived from UV exposed mushrooms on vitamin D status, particularly in recreationally active participants or athletes. Keegan et al. (2013) suggested that mushroom-derived vitamin D_2_ (2000 IU daily) demonstrated similar positive effects on 25(OH)D_TOTAL_ compared to D_3_, but did not include a placebo group [25]. Similarly, Urbain et al. (2011) found significant improvements in 25(OH)D_TOTAL_ for both a mushroom-derived and commercial vitamin D_2_ supplement (28,000 IU weekly) compared to placebo [27]. This potentially infers that higher doses, to that employed in the current study, may be required to significantly impact vitamin D status, although this has been contested elsewhere [20]. However, in this latter study [20], the processing of mushrooms may have significantly decreased vitamin D_2_ content, resulting in reduced daily intake. Therefore, encapsulated, dried, and pulverised extracts may increase mushroom-derived vitamin D_2_ bioavailability [25], with other studies indicating that daily doses > 600 IU may be required to elicit positive changes in vitamin D status [26].

A further consideration is that of individual 25(OH)D_TOTAL_ pre-intervention, and whether this limits or impacts the potential effectiveness of vitamin D_2_ supplementation. A recent meta-analysis [24] suggested that mushroom-derived vitamin D_2_ could be effective in raising 25(OH)D_TOTAL_ concentrations, but only when vitamin D status is classed as *insufficient to deficient (≤50 nmol·L*^*− 1*^*).* Previous research comparing mushroom-derived or commercial vitamin D_2_ over 6-weeks in healthy adults (mean 25(OH)D_TOTAL_ > 70 nmol·L^− 1^ at baseline) reported no overall treatment effects compared to control [51]. This was largely explained by increases in 25(OH)D_2_ coinciding with reductions in 25(OH)D_3_ of the same magnitude [51]. In the current study, 86% of participants were classified as having inadequate to deficient levels of 25(OH)D_TOTAL_. Baseline concentrations of 25(OH)D_TOTAL_ were statistically comparable between groups, however, the vitamin D_2_ group started with 57.8 ± 10.2 nmol·L^− 1^ which was + 11.7 nmol·L^− 1^ and + 6.0 nmol·L^− 1^ higher than the vitamin D_3_ and PL group, respectively. In agreement with Cashman et al. (2016) [24], this higher starting level of 25(OH)D_TOTAL_ could have potentially led to a non-significant interaction effect in our vitamin D_2_ group compared to the vitamin D_3_ group. It is noteworthy that 60% of the vitamin D_2_ group improved total vitamin D status from insufficient or worse (on average 38.2 ± 3.0 nmol·L^− 1^) to inadequate (62.1 ± 4.2 nmol·L^− 1^).

In the present study, each form of supplemented vitamin D had a direct and substantial positive impact on their corresponding 25(OH)D hydroxylated forms. Vitamin D_2_ supplementation significantly increased 25(OH)D_2_ concentration by + 347% over the 12-week intervention. The impact of vitamin D_3_ supplementation on 25(OH)D_3_ followed the same trend, with an overall improvement of + 111%. These results are in accordance with previous research, where 25(OH)D_2_ and 25(OH)D_3_ were measured independently [21, 30, 31, 35, 36], including studies using mushroom-derived vitamin D_2_ [1, 25, 26, 51], demonstrating similar bioavailability of both vitamins. As both 1,25(OH)_2_D_2_ and 1,25(OH)_2_D_3_ have been shown to have similar biological activity in vivo [8], both forms of supplementation likely have similar metabolic effects as demonstrated elsewhere [39, 52]. Therefore, it appears mushroom-derived vitamin D_2_ may offer an adjunct strategy, which is cost-effective and a more widely applicable food ingredient for populations (including vegans/vegetarians), with low vitamin D status in supporting their vitamin D intake.

Interestingly, however, in the vitamin D_2_ group, there was a significant − 42% reduction in 25(OH)D_3_ concentration from baseline to week 6, followed by a non-significant + 16% increase to week 12. This suppressing effect of vitamin D_2_ supplementation on 25(OH)D_3_ levels has been previously reported when commercially available forms of vitamin D_2_ were administered [34–36, 43], as well as mushroom-derived vitamin D_2_ [1, 26, 51]. This suppressing phenomenon could be responsible for the reduced efficacy of vitamin D_2_ in raising 25(OH)D_TOTAL_ compared to vitamin D_3_ [4, 24]. Although suppression mechanisms are not fully understood [4], chemically, vitamin D_2_ and D_3_ are structured differently [43]. This chemical variance could lead to a different affinity for the 25-hydroxylase receptors [30].

It has also been suggested that vitamin D_3_ hydroxylation may be impaired by vitamin D_2_ [35], as increases in 25(OH)D_2_ may lead to an increased catabolism of 25(OH)D_3_ [33]. However, this has been refuted by Stephensen et al. (2012) who argued that 25(OH)D_3_ catabolism should lead to increases in 24,25-dihydroxyvitamin D_3_ (24,25(OH)_2_D_3_), which were not detected in their study [51]. Additionally, it has been proposed that 25(OH)D_3_ has a greater binding affinity for the DBP compared to 25(OH)D_2_ in vitro [7]. A higher affinity for DBP would result in a greater concentration of circulating 25(OH)D_3_ and would decrease its rate of degradation, leading to a longer serum half-life of vitamin D_3_ and its metabolites [53]; and may also be associated with genotype. Indeed, depending on genotype for DBP, vitamin D_3_ supplementation has been shown to have differing effectiveness on raising 25(OH)D_TOTAL_ and 25(OH)D_3_ [45]. In contrast, efficacy of vitamin D_2_ supplementation was not affected by DBP genotype [45]. Therefore, mushroom-derived vitamin D_2_ supplementation should be less likely to be affected by DBP genotype.

In the current study, it is noteworthy that the PL group experienced a non-significant + 43% increase in 25(OH)D_3_ between week 6 to 12 (compared to + 23% and + 13% for vitamin D_2_ and D_3_ groups, respectively). As dietary intake of total vitamin D was maintained between groups across the intervention, the increases observed in 25(OH)D_3_, particularly in the vitamin D_2_ and PL groups, are most likely explained by a rise in the UV index (UVI), registered for Cambridgeshire, UK [54] towards the end of our intervention. Cardoso et al. (2017) reported that a higher UVI would impose a greater probability for endogenous production of vitamin D_3_. In their study, 25(OH)D_TOTAL_ started to increase once UVI was ≥3 [55]. Similar findings were reported in the current study, indicating a reduced effectiveness of vitamin D_2_ supplementation when this UVI threshold has been sufficiently exceeded.

It is important to note several limitations of the current study. Although dietary total vitamin D consumption remained consistent between groups across the intervention, it was noted that due to the sparsity of recorded vitamin D_2_ in food items [56], the analysis software only permitted quantification of overall dietary vitamin D. Therefore, it was assumed that any alterations in 25(OH)D_2_ were due to the vitamin D_2_ supplementation. Physical activity levels were not significantly different between the groups at any timepoint and did not change across the 12-week intervention for the vitamin D_2_ and D_3_ groups, but showed a significant decrease in the PL group based on their self-reported activity diaries. Activity diaries are not as reliable as objectively measured physical activity levels [57], which may have caused an over- and/or under-reporting throughout the intervention in the PL group.

Due to unforeseen delays, the study commenced at the end of January. Whilst findings may have been different if the study had commenced earlier, i.e. November to February, our results might have been impacted by increased sunlight exposure towards the latter half of the intervention. Furthermore, whilst we analysed blood samples for 25(OH)D_TOTAL_, 25(OH)D_2_ and 25(OH)D_3_, it would have been beneficial to also assess calcium and parathyroid hormone levels as parameters of bone [12] and vitamin D metabolism [35]. Finally, with a larger sample size, intervention groups could have been further divided based on vitamin D status classification to assess the impact of vitamin D_2_, with previous research suggesting that improvements may be more pertinent when participant baseline 25(OH)D_TOTAL_ levels are < 50 nmol·L^− 1^ [24, 44]. Future research should therefore consider effectiveness of mushroom-derived supplementation on vitamin D status in recreationally active individuals based on baseline levels and higher supplementation dose [58].

## Conclusion

The use of a UV-radiated mushroom food ingredient was effective in maintaining 25(OH)D_TOTAL_ in healthy, recreationally active volunteers. Mushroom-derived vitamin D_2_ powder may offer an adjunct strategy as a more cost-effective and widely applicable food ingredient for populations, including vegans and vegetarians, with low vitamin D status in supporting their vitamin D intake. Further research is required to find optimal dosages for daily mushroom-derived vitamin D_2_ supplementation. Consistent with the literature, vitamin D_3_ supplementation offers significant benefits when acute elevation in vitamin D status is warranted.

## Data Availability

The datasets used and/or analysed during the current study are available from the corresponding author on reasonable request.

## Abbreviations

25(OH)D: 25-hydroxyvitamin D
1,25(OH)_2_D: active 1,25-dihydroxyvitaminD
ANOVA: analysis of variance
AU: arbitrary units
CBAL: Core Biochemical Analysis Laboratory, Addenbrookes Hospital, Cambridge
DBP: vitamin D-binding protein
PL: Placebo
sRPE: session rating of perceived exertion
